# From the Cochrane Library: Interventions for Necrotizing Soft Tissue Infections in Adults

**DOI:** 10.2196/34578

**Published:** 2022-01-21

**Authors:** Hadir Shakshouk, Camille Hua, Brandon L Adler, Alex G Ortega-Loayza

**Affiliations:** 1 Department of Dermatology Oregon Health & Science University Portland, OR United States; 2 Department of Dermatology Hôpital Henri Mondor Créteil France; 3 Department of Dermatology Keck School of Medicine University of Southern California Los Angeles, CA United States

**Keywords:** necrotizing soft tissue infections, therapy, intervention, systematic review, infections, management, evidence-based medicine, dermatology, skin infection

Necrotizing soft tissue infections (NSTIs) refer to severe life-threatening bacterial infections involving the dermis, subcutaneous tissue, fascia, or muscle. NSTIs can lead to serious morbidities and mortality. Diagnosis can be challenging, and a high index of suspicion is required. Useful clues include pain out of proportion to skin findings, manifestations of systemic toxicity, and lack of response to systemic antibiotics. While crepitus, hemorrhagic bullae, skin necrosis, skin anesthesia, and symptoms of sepsis are typical of NSTIs, confirming the diagnosis requires surgical exploration [1].

Management entails early surgical debridement coupled with empiric broad-spectrum intravenous antibiotics against both aerobic and anaerobic organisms in addition to intensive care support. Tissue hypoxia and necrosis induced by NSTIs limit the efficacy of systemic antibiotics, rendering surgical debridement the mainstay treatment [1].

A Cochrane review [1] investigated available interventions for NSTIs. The inclusion criteria specified randomized controlled trials of medical or surgical interventions in hospital settings for adults with NSTIs. Adjunctive hyperbaric oxygen therapy was addressed in a prior Cochrane review [2]. The primary outcome measures were mortality within 30 days and occurrence of serious adverse events, whereas the secondary outcomes were survival time as well as long‐term morbidity assessed via the Functional Impairment Scale [1].

The authors identified 3 trials comprising 197 participants (n=117, 62% men) with a mean age of 55 years. In all trials, patients received the standard of care (ie, surgical debridement, empiric antibiotics, and intensive care support). The used empiric antibiotics were vancomycin, clindamycin, ciprofloxacin, and piperacillin-tazobactam [1]. One trial compared 2 antibiotic treatments, moxifloxacin 400 mg once daily and amoxicillin‐clavulanate 3 g three times daily for at least 3 days, followed by 1.5 g three times daily [3]. Another trial evaluated the novel drug AB103, studied also for sepsis, which impairs T-cell activation by blocking the binding of superantigen exotoxins to the CD28 receptor on T‐helper1 lymphocytes [4]. Two doses (0.5 mg/kg and 0.25 mg/kg) were investigated against the placebo. The third trial assessed intravenous immunoglobulin at a dose of 25 g/day, given for 3 consecutive days, versus a placebo [5].

In all trials, no difference was detected between groups regarding the primary outcome measures. The quality of evidence was assessed as low to very low; this implies uncertainty in these results. Adverse events, secondary outcomes, and median survival times are summarized in Table 1. None of the trials assessed long-term morbidity as defined in the review protocol [1].

**Table 1:** A summary of trials included in the Cochrane review [1].

Characteristic			Trials		
			MXF^a^ vs AM-CL^b^, Vick-Fragoso et al [3]	AB103 vs placebo, Bulger et al [4]	IVIG^c^ vs placebo, Madsen et al [5]
Groups			1. MXF 400 mg once daily 2. AM-CL 3 g three times daily for at least 3 days followed by 1.5 g three times daily	1. AB103 0.5 mg/kg 2. AB103 0.25 mg/kg 3. Placebo Single intravenous dose within 6 hours after diagnosis	1. IVIG 25 g/day for 3 consecutive days 2. Placebo
Participants, n			54 (MXF group: n=36; AM-CL group: n=18)	43 (AB103 group: n=32; placebo group: n=11)	100 (IVIG group: n=50; placebo group: n=50)
Overall risk of bias			High (attrition, imbalance, performance, detection)	Moderate (attrition)	High (attrition, imbalance)
**Primary outcomes**					
** **	**Mortality within 30 days**		No difference (RR^d^ 3.00, 95% CI 0.39-23.0)	No difference (RR 0.34, 95% CI 0.05-2.16)	No difference (RR 1.17, 95% CI 0.42-3.23)
		Certainty of evidence	Very low	Very low	Low
** **	**Proportion of patients who experienced serious adverse events**		Not specified; no difference (RR 0.63, 95% CI 0.30-1.31)	Not specified; no difference (RR 1.49, 95% CI 0.52-4.27)	Acute kidney injury, allergic reactions, aseptic meningitis, hemolytic anemia, thrombi, and infections; no difference (RR 0.73, CI 95% 0.32-1.65)
		Certainty of evidence	Very low	Very low	Low
**Secondary outcomes**					
	Survival time (median time of death)		Shorter in the MXF group (10.5 days vs 42 days); no statistical analysis was possible	Not specified	Shorter in the IVIG group (25 days vs 49 days); no statistical analysis was possible
	Assessment of long‐term morbidity		Not specified	Not specified	No difference in the median physical component summary scores between groups (mean adjusted difference 1, 95% CI 7-10; *P*=.81)

^a^MXF: moxifloxacin.

^b^AM-CL: amoxicillin‐clavulanate.

^c^IVIG intravenous immunoglobulin.

^d^RR risk ratio.

The quality of the evidence was negatively impacted by attrition bias, indirectness due to the lack of a definition of NSTIs, small sample size, and underpowered analysis. The lack of high-quality evidence for this serious condition necessitates the need for larger, well-designed studies. A recent randomized controlled trial evaluated the efficacy of AB103 0.5 mg/kg versus placebo when administered within 6 hours of NSTI diagnosis [6]. No significant improvement was found in the primary composite endpoint (28-day mortality, number of debridements, amputations after the first operation, and resolution of organ dysfunction) in intention to treat whereas there was in the per-protocol population [6]. Given the rarity of NSTIs and their complex diagnosis and management, prospective registries are encouraged to provide evidence for effective therapeutic approaches to improve morbidity and mortality.

## Abbreviations

NSTI: necrotizing soft tissue infection
